# Radiotherapy–Immunotherapy Combination: How Will We Bridge the Gap Between Pre-Clinical Promise and Effective Clinical Delivery?

**DOI:** 10.3390/cancers13030457

**Published:** 2021-01-26

**Authors:** Erminia Romano, Jamie Honeychurch, Timothy M. Illidge

**Affiliations:** 1 Division of Cancer Sciences, Faculty of Biology, School of Medical Sciences, Medicine and Health, University of Manchester, Manchester M13 9PL, UK; erminia.romano@manchester.ac.uk (E.R.); jamie.honeychurch@manchester.ac.uk (J.H.); 2 Manchester Academic Health Science Centre, NIHR Biomedical Research Centre, The Christie NHS Foundation Trust, Manchester M20 4BX, UK

**Keywords:** radiotherapy, immunotherapy, immuno-oncology agents, radiotherapy dose, fractionation schedule, treatment field, tumor volume, administration site/route, administration sequencing

## Abstract

**Simple Summary:**

Radiotherapy is an important component of cancer treatment, given to around half of all cancer patients. Radiotherapy is known to be very effective at directly killing cancer but, until recently, the important effects that radiotherapy has on the surrounding immune cells were not widely appreciated. Over the last decade, immunotherapy approaches have made a major breakthrough in cancer treatment, and now play an important part of routine cancer care. Given that both radiotherapy and immunotherapy can stimulate anti-tumor immune response, it is logical to combine these approaches to try and improve anti-tumor immunity and cancer outcomes further. This review assesses the important clinical questions that need to be addressed to successfully combine radiotherapy and immunotherapy treatments by rethinking approaches to the delivery of radiotherapy, as well as the optimal type and scheduling of immunotherapy.

**Abstract:**

Radiotherapy (RT) is highly effective at directly killing tumor cells and plays an important part in cancer treatments being delivered to around 50% of all cancer patients. The additional immunomodulatory properties of RT have been investigated, and if exploited effectively, have the potential to further improve the efficacy of RT and cancer outcomes. The initial results of combining RT with immunomodulatory agents have generated promising data in pre-clinical studies, which has in turn led to a large number of RT and immunotherapy clinical trials. The overarching aim of these combinations is to enhance anti-tumor immune responses and improve responses rates and patient outcomes. In order to maximize this undoubted opportunity, there remain a number of important questions that need to be addressed, including: (i) the optimal RT dose and fractionation schedule; (ii) the optimal RT target volume; (iii) the optimal immuno-oncology (IO) agent(s) to partner with RT; (iv) the optimal site(s)/route(s) of administration of IO agents; and finally, the optimal RT schedule. In this review, we will summarize progress to date and identify current gaps in knowledge that need to be addressed in order to facilitate effective clinical translation of RT and IO agent combinations.

## 1. Introduction

**Figure 1:**
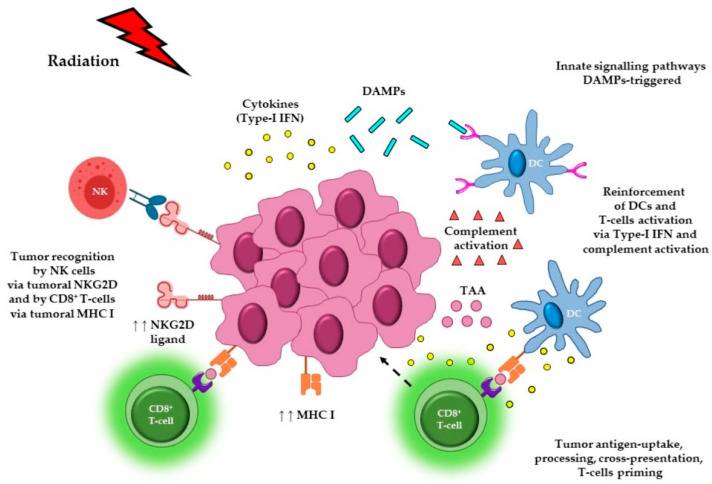
Immunomodulatory effects of radiotherapy. Local radiation to the tumor can elicit immunogenic cell death, leading to the release of cytokines and DAMPs that in turn trigger innate signaling pathways. These signals favor the recruitment of APCs such as DCs, promote uptake of dying tumor cells, and enhance the processing of TAAs and cross-presentation of antigenic-peptides, via MHC I, to CD8^+^ T cells. Cross-presentation of tumor-antigens can lead to the priming of tumor-specific T lymphocytes, which can then traffic back to the tumor site, infiltrate into the tumor, and potentially exert cytolytic effector activity. Radiation can also induce the release of type-I IFN from both cancer and immune cells, as well as trigger complement activation, which can reinforce both DC and T cell activation. RT can also cause upregulation of MHC I, the adhesion molecule, ICAM, and the membrane protein NKG2D type II (ligand), that ultimately enhance tumor recognition and killing by T cells and NK cells, respectively. Abbreviations. DAMPs: damage associated molecular patterns; APCs: antigen presenting cells; DCs: dendritic cells; TAAs: tumor-associated antigens; MHC I: major histocompatibility complex class I; IFN: interferon; NKG2D: natural killer group 2D; NKs: natural killer cells. Figure created with the aid of biorender.com and https://smart.servier.com/.

Radiotherapy (RT) is an important component of standard of care for approximately 40–50% of all cancer patients and is used with both curative and palliative intent [1]. The most extensively investigated mechanism of action of ionizing radiation is the induction of irreversible damage to DNA and other macromolecules, which leads to failure of cancer cells to replicate and ultimately to cellular death. In addition to the direct tumoricidal effects of RT, emerging evidence suggests that RT can have important immune modulatory effects [2,3]. These immunomodulatory effects are observed both locally, in the tumor microenvironment (TME), and systemically, in regional lymph nodes and blood [4,5]. Local radiation has the potential to elicit an immune-stimulatory form of cell death, termed immunogenic cell death (ICD) [6]. This process leads to the release of cytokines and damage associated molecular patterns (DAMPs), a set of distinct molecular signals that trigger innate signaling pathways, similar to viral pathogens [6]. These signals favor the recruitment of antigen presenting cells (APCs), enhancing their phagocytic activity, processing of tumor-associated antigens (TAAs), and cross-presentation of antigenic peptides on major histocompatibility complex class I (MHC I). Cross-presentation of tumor antigens can lead to subsequent priming and trafficking of tumor-specific T lymphocytes into the TME [6]. Complement activation and production of type-I IFN (interferon) can also occur, reinforcing both dendritic cell (DC) and T cell activation. RT can also induce upregulation of MHC I, the intercellular adhesion molecule-1 (ICAM-1) and membrane protein NKG2D type II on cancer cells, that enhance recognition and cytolysis of tumor by T cells and natural killer (NKs) cells, respectively [1] (Figure 1). Other cells present in the TME, that may have a profound influence on outcome and response to RT, include cancer-associated fibroblasts (CAFs). These cells support tumor immune evasion by affecting the features and recruitment of both myeloid and lymphoid cells, creating a physical-chemical immune barrier, and ultimately, an immunosuppressive TME. Given their immunogenic role and their ability to exchange signals with both cancer and immune cells, CAFs represent good candidates for the optimization of therapeutic strategies. Although a detailed description of CAF biology is beyond the scope of this review, the subject is covered in detail elsewhere [7,8,9,10].

T cell activation can also contribute to immune responses outside the radiation field, likely responsible for inducing the so-called “abscopal effect”, as shown by studies demonstrating the requirement of T cells in mediating the systemic response prompted by RT [11,12]. However, in clinical practice, such “abscopal” responses following RT to the primary tumor, leading to clinically meaningful responses in sites of metastatic cancer, are an extremely rare phenomenon [5]. Nevertheless, there is now considerable pre-clinical evidence to support the concept that RT can contribute positively to the priming of effector T cells, leading to anti-tumor immune responses. However, in contrast to these immunostimulatory effects, RT can also induce immune-suppressive responses by increasing the ratio of radio-resistant regulatory T lymphocytes (T_regs_), or by promoting immunosuppressive immune effector cells, such as macrophages and other myeloid derived cells [1]. Given these immunosuppressive effects, it is perhaps not surprising that single treatment RT is rarely able to promote significant systemic anti-tumor immune responses. However, manipulating the potent immune-modulatory properties of RT through combination with immuno-regulatory agents provides a real opportunity to further improve the efficacy of RT and cancer outcomes [13,14].

The initial pivotal studies using the immune check-point inhibitors (ICIs) anti-Cytotoxic T Lymphocyte-Associated protein-4 (CTLA-4) and anti-Programmed Death/Ligand-1 (PD-1/PD-L1) led to durable remissions in a minority of patients with incurable metastatic cancers, such as metastatic malignant melanoma and non-small cell lung cancer (NSCLC) [15,16,17,18]. This striking clinical efficacy led to the regulatory approval of these ICIs, and established immunotherapy as another effective form of cancer treatment. However, despite this exciting and breakthrough therapy, only a minority of solid cancer patients respond to ICIs [16].

Given the immunomodulatory effects of RT, this provides a clear rationale for combining RT with ICIs to enhance the overall efficacy. Indeed, the early pre-clinical tumor models provided encouraging support for the potential of RT and immunotherapy combinations. These initial studies led to the initiation of large numbers of clinical studies and considerable optimism that RT and IO agent combinations would improve cancer survival and transform the management of cancer [17,18,19,20,21].

However, despite a number of important breakthroughs in the field, several key questions need to be addressed if this initial optimism of the pre-clinical data is to be effectively translated into clinical trials that lead to improved clinical outcomes:

- What is the optimal radiation dose and fractionation to induce the most effective anti-tumor immune responses?
- What is the optimal tumor volume and RT treatment field to elicit the most effective tumor control?
- Does schedule of RT–IO agents impact on the induction of systemic anti-tumor immunity, and does this vary with the IO agent and tumor type? Moreover, does the route of delivery (intra-tumoral versus intravenous) of the IO agent(s) influence the generation of both local and abscopal responses in combination with RT?

## 2. The Effect of Radiotherapy Dose and Fractionation on Anti-Cancer Immune Responses

The optimal radiation dose and fractionation schedule required to generate effective local and systemic anti-tumor immune responses in combination with IO agents, remains underexplored and requires further investigation. The evaluation of different RT doses and fractionation regimens have been largely explored using murine tumor models, comparing single high dose RT versus fractionated short course of RT doses. These pre-clinical studies confirm the potential importance of RT dose and fractionation in altering the immune contexture within the TME [22]. However, these murine tumors and RT dose fractionation may not be representative of the clinical situation, when considering the heterogeneity of patient tumors. Furthermore, the protracted “radical” doses over 6–7 weeks using 1.8–2.0 Gy per fraction have not been tested in the pre-clinical setting.

One such study investigated a single high dose of 30 Gy in CT26 and MC38 colon cancer models, which induced durable systemic anti-tumor response resulting in long tumor control [23]. Interestingly, a single 30 Gy dose led to infiltration of CD8^+^ T cells into the TME that was not observed after treatment with a fractionated regimen of 10 daily doses of 3 Gy each. The immunosuppressive TME characterized by myeloid derived suppressor cells (MDSCs) was reversed with the assistance of cross-presenting CD8^+^ DCs, CD4^+^ T cells, and IFN-γ produced by CD8^+^ T cells, ultimately inducing durable tumor control. In contrast to these data demonstrating the superiority of a large single dose of radiotherapy, other published data suggest that hypo-fractionated radiation schedules are better able to generate local and abscopal immune responses, both alone and in combination with ICIs. One such study, characterizing the effects of different fractionation protocols, demonstrated that 18 × 2 Gy and 3 × 8 Gy, given alone, induced enhanced tumor growth delay compared to a single dose of 16.4 Gy RT, in mouse models of colon cancer (CT26) and melanoma (B16-F10) [24]. Interestingly, the authors reported a differential effect on the involvement of distinct immune cells populations according to the RT schedules used. In particular, a predominant lymphoid response (CD8^+^ T cells and T_regs_) was induced by 3 × 8 Gy and by a single fraction of 16.4 Gy. In contrast, a predominantly myeloid subset cell response (MDSCs and M2-macrophages) was initiated by 18 × 2 Gy. Moreover, differences in tumor control were also observed when combining these RT regimes with different ICIs. The use of 3 × 8 Gy in combination with immunomodulation directed at the PD-L1 and T cell immunoglobulin and ITIM domain (TIGIT) resulted in the best tumor control [24].

Dewan et al. reported that 3 × 8 Gy and 5 × 6 Gy, in contrast to a single dose of 20 Gy RT, in combination with anti-CTLA-4 mAb (monoclonal antibody), resulted in enhanced tumor growth inhibition in breast (TSA) and colon (MCA38) carcinoma models, at both primary and secondary tumors, with induction of an abscopal effect [25]. Furthermore, they showed an IFN-γ mediated role for CD8^+^ T cells in tumor inhibition at the secondary sites. The differential role of these RT doses and fractionation regimens in combination with anti-CTLA-4 mAb, in generating systemic anti-tumor immunity, is particularly interesting, given that each of these radiotherapy schedules, were equally effective in delaying the growth of the irradiated primary tumors [25]. Abscopal responses have also been observed in a murine mammary model (TSA), following treatment with 3 × 8 Gy RT, but not a single dose of 30 Gy, in combination with anti-CTLA-4 mAb [26]. Both RT schedules were equally ineffective in controlling the tumor growth when delivered as single agent therapy, and durable tumor control was only observed in combination with anti-CTLA-4 mAb. Interestingly, the authors also observed improved control of the irradiated tumor when using a single RT dose of 8 Gy with anti-CTLA-4 mAb. Furthermore, following CD8^+^ T cell depletion, tumor regression and the abscopal response were lost, suggesting the essential role played by these cells in this context [26].

The induction of an abscopal response has also been reported for anti-PD-1 mAb in combination with hypo-fractioned regimen (3 × 9.18 Gy given in three or five days, or doses of 5 × 6.43 Gy in 10 days) in metastatic models of melanoma (B16-CD133) and breast cancer (4T1) [27]. In these settings, when anti-PD-1 was given together with RT, superior tumor growth inhibition of primary and secondary (non-irradiated) tumors and overall survival were observed, compared to RT or anti-PD-L1 given alone. Furthermore, combination therapy led to a CD8^+^ T cell-mediated immune response [27]. A similar systemic response has also been showed in colorectal carcinoma (MC38), when combining anti-PD-1 with a fractionation regimen of 3 × 8 Gy [28].

Deng et al. reported effective tumor control at primary tumor sites and non-irradiated distant tumors with durable T cell immunity, when combining anti-PD-L1 mAb with a single dose of 12 Gy or 20 Gy in breast (TUBO) and colon (MC38) cancer models, respectively [29]. In particular, they showed an immune suppressive response following irradiation, characterized by increased expression of PD-L1 within the TME, subsequently counteracted with the combinatorial approach. They also reported the crucial role played by CD8^+^ T cells in this scenario and a reduction in the local accumulation of tumor-infiltrated MDSCs due to tumor necrosis factor-alpha (TNF-α) produced by these T lymphocytes [29].

Finally, studies also support the benefit of using lower doses of RT in a fractionated regimen in combination with ICIs, as demonstrated by Dovedi et al. In a mouse model of colon carcinoma (CT26) treated with 10 Gy RT delivered in five fractions of 2 Gy, RT resulted in the activation of CD8^+^ T cells, producing IFN-γ that led to adaptive upregulation of PD-L1 within the TME. The concomitant administration of an anti-PD-1/PD-L1 antibody was able to overcome this adaptive resistance and led to the generation of effective and durable CD8^+^ T cell-mediated immune response, resulting in local and distal tumor control, providing long-term survival and protection against tumor re-challenge. Furthermore, both resident and infiltrating T cells were responsible for tumor regression at the locally irradiated sites, and for induction of regression of the out of field lesions [30,31]. All of the published studies emphasize the importance of CD8^+^ T cells in tumor control.

Uncovering the impact that RT dose and fractionation has on immune-modulatory effects is essential for clinical translation. Currently, there is no consensus regarding the optimal dose and fraction of RT to use to elicit an effective and durable anti-tumor immune response. Consequently, a wide range of different RT doses and fractionation regimens are currently being tested in the clinic. One of the established standards for RT used with “radical” intent is (1.8–2.0 Gy/fraction) daily for 6–7 weeks. However, such protected low dose per fraction regimens may not be optimal for the induction of anti-tumor immune responses, and hypo-fractionated regimens (3–20 Gy/fraction), given once per day and in a shorter timeframe, may be better able to induce anti-tumor immunity [32].

Based on the reassuring initial patient-safety data, several clinical trials have started, combining hypo-fractionated or fractionated radiation with anti-PD-1/PD-L1 mAb, or anti-CTLA-4 mAb or a combination. Encouraging results have been reported for melanoma patients treated with ipilimumab (anti-CTLA-4 mAb) in combination with 24 Gy delivered in three fractions [33], 28.5 Gy in three fractions over a window of seven days [34], or ≤3 Gy in multiple fractions [35]. Similar results were observed in NSCLC patients, experiencing abscopal effect after ipilimumab in combination with RT at 28.5 Gy given in three fractions over a period of seven days [36], or with a 6 × 5 Gy daily fraction regimen [37]. The study by Formenti et al. in NSCLC patients who had failed to respond to ICI alone or in combination with chemotherapy, reported a systemic anti-tumor response mediated by T cells when treated with anti-CTLA-4 mAb in combination with either 5 × 6 Gy or 3 × 9.5 Gy. Although objective responses were observed in only 18% of patients, 31% experienced disease control. This study demonstrates that a hypo-fractionated RT regimen, in combination with anti-CTLA-4 mAb, resulted in an increase in interferon-β (IFN-β) in the serum after RT, together with changes in T cell clonality [38]. These interesting data confirm the mechanistic pre-clinical findings indicating a role for IFN-β, in the recruitment and activation of DCs that result in the priming of a CD8^+^ T cell-mediated tumor response [26].

In summary, the current pre-clinical and clinical reports highlight the diversity of responses in different models and tumor types and emphasize the importance of carefully conducting studies aimed at further investigating RT dose and fractionation in different tumor types, to optimize approaches.

## 3. The Potential Effects of Radiotherapy Treatment Volumes on Immune Responses and Tumor Control

The volume of tumor irradiated, and the size of treatment field, are highly likely to influence the type and magnitude of the local and systemic anti-cancer immune response.

### 3.1. Irradiation to Draining Lymph Nodes

**Figure 2:**
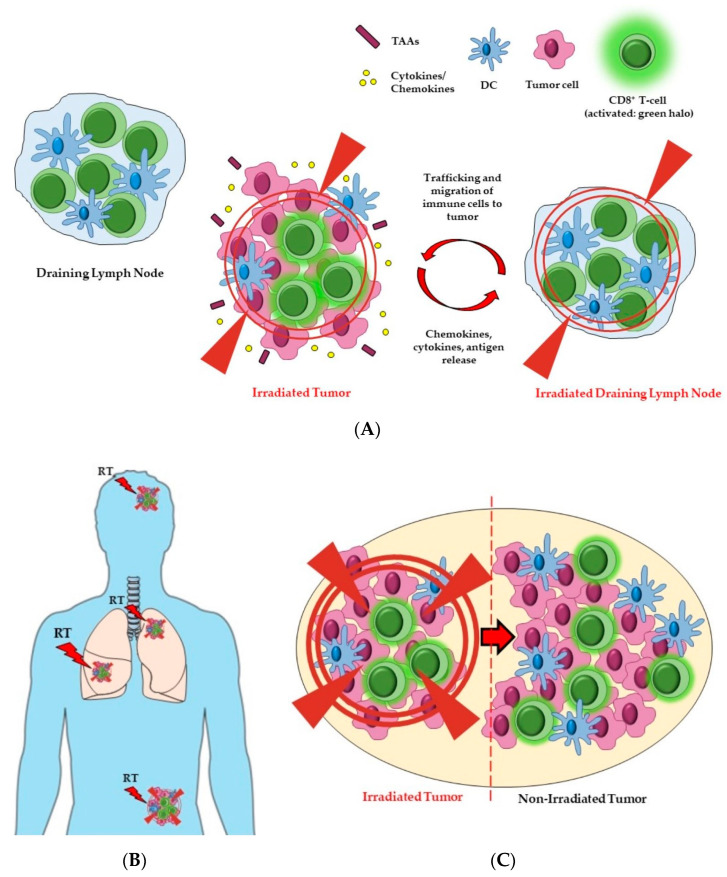
The potential effects of radiation field and volume on immune responses and tumor. The size of the treatment field and the volume of the tumor irradiated can influence both local and systemic anti-cancer immune responses. (**A**) Including the DLNs (right side) in the radiation field could affect the balance of cytokines and chemokines which, together with the release and processing of tumor antigens, leads to trafficking and migration of immune cells (i.e., CD8^+^ T cells and DCs) to the tumor. Cancer cell eradication will be mediated by activated (green halo) CD8^+^ T cells. However, RT directed towards DLNs may also have cytotoxic effects on lymphocytes, hence impeding an effective anti-tumor immune response. (**B**) The irradiation of oligometastases, in addition to the primary tumor, might favor the expression or release of greater amounts and/or new tumor antigens, in a way that is proportional to the tumor burden. Multi-site RT may therefore lead to the priming and subsequent infiltration of immune cells into the different lesions (sensitization of the tumors to immune infiltration). (**C**) Partial tumor irradiation might be a way to indirectly control the whole tumor, by generating immune activation starting from the hemi-irradiated part. For example, activated CD T cells (shown in green) may migrate from the field of RT into the remainder of the tumor, whereby they may display anti-cancer activity in the non-irradiated part. Abbreviations. DCs: dendritic cells; TAAs: tumor-associated antigens. Figure created with the aid of biorender.com and https://smart.servier.com/.

In radical treatments, the clinical target volume (CTV), may, in some clinical situations, encompass not only the surrounding normal tissue, but additionally, the regional draining lymph nodes (DLNs) (Figure 2A). The strategy of elective nodal irradiation (ENI) is aimed at targeting possible subclinical nodal micro-metastasis, in the treatment of localized cancers. However, the delivery of RT to the DLNs could potentially have profound effects on the ability to generate a systemic immune response. In addition to the local effects of RT on immune effector cells within the DLN, modern multiple field IMRT (intensity-modulated radiation therapy) may have important depleting effects on circulating lymphocytes which are more sensitive to the cytotoxic effects of RT compared to macrophages, myeloid cells, and DCs. Furthermore, it is possible that regional nodal irradiation and multi-field IMRT plans may compromise the proliferation of T cells and their priming in the lymph nodes [39]. Consequently, it is important to reconsider the effects of irradiation on the DLNs and how this may potentially modulate the adaptive immunity.

Initial pre-clinical studies have demonstrated that DLNs are essential for the accumulation of antigen-specific cytotoxic T lymphocytes (CTLs) induced by RT [40]. Moreover, when RT is combined with anti-PD-1 mAb, tumor antigen-specific central memory cells increase in the DLNs [41] (see Table 1a for further details). A study on colorectal cancer (MC38) and melanoma (B16-F10) from Marciscano et al. (on localized and non-metastatic tumors), demonstrated that sparing DLNs from RT can be beneficial for combinatorial approaches with anti-CTLA-4 mAb and anti-PD-1 mAb, because the irradiation negatively affected the balance of chemokines recruiting effector T cells into the TME, hence reducing antigen-specific immune infiltration. Combination with ICIs in this setting improved survival, because it increased the intra-tumoral density and ratio of CD8^+^ T cells versus T_regs_ [42]. This study informs the rationale and importance of new RT and ICI combination trials investigating whether excluding DLNs from the target volume of irradiation enhances anti-tumor immune responses. Further studies using a melanoma model (B16-F10 modified with a viral glycoprotein to facilitate the identification of tumor specific T cells) showed that local tumor irradiation improved abscopal response and stimulated the proliferation of both total CD8^+^ and stem-like CD8^+^ T cells in the DLNs. However, when both the tumor and the DLNs were irradiated, the abscopal response was reduced, and this was associated with a reduction in the number of tumor specific CD8^+^ T cells, as well as stem-like CD8^+^ T cells, in both irradiated and not-irradiated tumors. These data suggest that DLNs might mediate the abscopal response, and potentially serve as a pool of stem-like CD8^+^ T cells, which can expand and migrate to populate the tumor [43].

### 3.2. Irradiation to Single or Multiple Tumor Sites (Oligometastases)

For palliative treatments, where there may be multiple sites of metastatic disease, it is unknown whether irradiation to a single tumor, or inclusion of the metastatic sites, would be better able to generate an anti-tumor immune response (Figure 2B). Here, the working hypothesis is that the antigen load released from dying tumor cells that is required to generate an anti-tumor immune response following RT is proportional to the number of sites or tumor volume treated. This hypothesis requires further testing in pre-clinical models and well-designed clinical studies. Indeed, there is currently limited data regarding whether irradiating the primary tumor on more than one site, so called “oligometastases” (fewer than five metastatic lesions), results in improved anti-tumor immune responses and tumor control.

One such study in the B16-F0 melanoma model showed that, in mice treated with localized RT, there was an increase in the antigen-presentation within the DLNs and in T cells secreting IFN-γ, upon tumor antigen stimulation within these tumor DLNs. Furthermore, this response was associated with trafficking to the tumor site of immune cells, including tumor infiltrating lymphocytes (TILs) secreting IFN-γ and having lytic activity [44]. Conversely, work in the pancreatic model Panc02 revealed that irradiating a second tumor significantly reduced the growth of further malignancy at a non-treated third site [45]. Other studies, using breast (TSA) and colon (MCA38) cancer models, showed inhibition of the metastatic process following RT treatment of the primary tumor in combination with anti-CTLA-4 [25,46].

In the clinic, there are a limited number of studies that resulted in a modest effect following irradiation of a single metastatic lesion. Kwon et al. reported negative results when only one bone metastasis was treated with RT and anti-CTLA-4 mAb, in patients with metastatic castration-resistant prostate cancer [47]. In contrast, irradiating multiple lesions promoted promising effects when RT was combined with anti-PD-1 mAb in patients with a range of metastatic solid tumors [48]. Another report indicated that, in the context of oligometastases, ablative doses of RT together with IO agents improved the control rates of cancers including melanoma, NSCLC, and kidney cancer [49]. A study from Gomez et al. supported the potentially curative approach of irradiating all lesions as standard of care for patients with oligometastatic NSCLC [50].

### 3.3. Partial Tumor Irradiation

The optimal treatment volume remains uncertain, and questions regarding the tumor volume to treat include: (i) Does the whole macroscopic tumor need to be treated to the full dose? (ii) Would treatment to a proportion of the tumor with a high dose and other areas with a lower dose enhance immune cell infiltration? (iii) Would the omission of RT from a part of the tumor still result in sufficient antigen release and/or more favorable immune effect changes in the TME, with recruitment of T cells (so called ‘RadScopal’ effect) (Figure 2C) to generate a more effective systemic anti-tumor immune response [51]?

The ‘RadScopal’ effect arises as a consequence of radiation directed to part of the primary tumor, and may be accompanied by low-dose RT towards secondary lesions. It has been proposed that this double form of radiation, combined with ICIs, might be able to modulate the TME of both primary and secondary tumors, maximizing the anti-tumor immune responses [51]. Barsoumian et al. investigated the priming of T cells in the Lewis lung carcinoma (LLC) mouse model using high-dose RT to a higher tumor burden. They further investigated the combination of low-dose RT to the metastatic sites, in order to modulate the stroma. Interestingly low-dose RT enhanced the anti-tumor responses to ICIs (anti-PD-1 and anti-CTLA-4), promoting M1-polarization of macrophages, NK infiltration, and reduction in transforming growth factor beta (TGF-β) levels. These data suggest that low-dose RT can reprogram the TME, improve the infiltration and function of effector immune cells in the secondary tumors, and are able to overcome the inhibitory effects of the tumor stroma. Finally, the combination with the two ICIs could further increase and prolong the systemic effects of RT, via T_regs_ blocking and attenuation of T cell exhaustion [51].

A potentially important development is the use of spatially fractionated radiotherapy (SFRT) or GRID irradiation. SFRT irradiation allows the delivery of a relatively large (15–20 Gy) but heterogeneous single dose, through a perforated block that ultimately fractions the beam into smaller units, while sparing the skin and tissues between adjacent holes from side effects. In this way, larger doses can be delivered in a single fraction (or a few weekly fractions) and ultimately induce the damage/killing of the cells of the shielded non-irradiated areas within the tumor (bystander effect). Furthermore, emerging pre-clinical studies suggest that SFRT might also prompt immunomodulatory effects [52].

Experiments on mouse models of breast (67NR) and lung (LLC) cancer, showed tumor eradication when a single dose (10–20 Gy) was delivered to half a tumor, similarly to the fully irradiated one. The immune response elicited was mediated by CTLs that infiltrated the tumor via a mechanism that appeared to be mediated by vascular adhesion molecules (ICAMs). Interestingly, when the exodus of CD8^+^ T cells from LNs was inhibited, there were no effects on tumor response, suggesting that the source of infiltrating T lymphocytes were not the DLNs, but most likely the targeted section of the hemi-irradiated tumor [53]. Overall, this study suggests that partial radiation alone can be immune-stimulatory and indirectly control tumor growth via immune activation, inducing abscopal effect in the contralateral tumor.

The RadScopal effect, has been reported in patients receiving RT alone or in combination with ICIs, providing complete tumor responses, despite only partial tumor irradiation. Lemons et al. investigated the combination of the anti-PD-1 mAb Pembrolizumab with multi-organ site ablative radiation therapy (MOSART), reporting comparable local disease control for both the large partially irradiated tumors, as well as the small lesions included in the radiation volume targeted [54].

There are few reports of the immunomodulatory effects of low-dose irradiation on out-of-field responses [55,56]. Barsoumian and colleagues reported a phase II clinical trial involving patients with metastasis to the lungs or liver and that were treated with 50 Gy RT delivered in four fractions targeting the tumor lesions, with concurrent or sequential administration of ipilimumab. Intriguingly, a 40% response rate was observed in the proximal, but not directly irradiated lesions, in combination with ipilimumab. A 10% response rate was seen in the distant lesions that were not exposed to RT [55]. In another clinical study investigating the effects of low-dose radiation in combination with high doses plus immunotherapy in metastatic cancers, patients received low-dose RT (1–20 Gy, either as direct treatment or as scatter from high-dose radiation) to the metastasis, together with high dose radiation to another lesion, in combination with ICIs (anti-CTLA-4 and anti-PD-1 mAbs). Responses for both non-irradiated and low-dose irradiated lesions were observed, suggesting that an enhanced immune response and abscopal effects can take place when treating with low-dose radiation, which may enhance responses in secondary tumors [56].

In summary, DLN irradiation involves a potentially delicate balance between the beneficial anti-cancer cytotoxic effects of RT in clearing micro-metastatic disease, versus the RT-induced depletion of important immune effector cells, which may result in impaired anti-tumor immune responses. In addition to these considerations regarding lymph nodes irradiation, there may be organ-specific differences in the TME that may influence the nature and quality of the immune response, both with RT alone and in combination with IO agents. Finally, regarding the volume of the tumor field irradiated, larger volumes may result in the increased release of DAMPs, tumor antigens, and a more robust anti-tumor immune response. However, an increase in the total field irradiated might also lead to the depletion of immune effector cells that may be important in long-term tumor control. Therefore, reduced RT treatment volumes may be advantageous in enhancing anti-tumor immune responses, but this requires further rigorous and careful clinical investigation. In contrast to potentially reducing treatment volume with partial irradiation of large tumors, in the clinical situation of multiple metastases, irradiation of a single lesion may not be sufficient to generate the release of enough tumor-associated antigens to generate a robust anti-tumor immune response. Therefore, delivery of RT to multiple metastatic lesions could be preferable to enhance immune infiltration to multiple tumor sites, especially when combined with immunomodulatory agents. Finally, the immune contexture of the TME may play an important role in determining the tumor volume to target, which may vary amongst different cancer types. Therefore, studies aimed at the characterization of the tumor and its immunological context before and after RT treatment, comparing different targeted tumor volumes and fields, might help in identifying any possible correlation between optimal tumor size(s) and field(s) to target, and the most effective anti-tumor response, in combinatorial approaches. This local analysis should be complemented with a systemic one, in order to assess potential occurrence of abscopal effect.

## 4. Combining Radiotherapy with Immunomodulatory Agents

The breakthrough of ICIs and the subsequent emergence of other IO agents has revolutionized the treatment of cancer. So far, focus has been on co-inhibitory receptors/ligands to overcome immune suppression, e.g., anti-CTLA-4, anti-PD-1, anti-PD-L1, and targeting co-stimulatory molecules to induce activation pathways, e.g., anti-CD40. Despite the antibodies approved by the Food and Drug Administration (FDA) and currently used in the clinic, only the minority of patients respond to ICIs, and if they do, they often develop secondary resistance and/or show immune related adverse events (irAEs) [57]. For this reason, more attention is increasingly paid to the potential targeting of other molecules, to use in combinatorial approaches. In this scenario, RT is considered a good partner, given its immunomodulatory properties and hence its possible enhancer/synergistic anticancer activity. Table 1 gives an overview of the molecules currently targeted in combination with RT, in pre-clinical (Table 1a) and clinical studies (Table 1b), reporting the immune-related events observed in each study.

## 5. Scheduling and Site of Delivery of IO Agents in Combination with RT

### 5.1. Scheduling

The overall efficacy of RT with IO agents may be influenced by a number of important parameters, including: (i) the scheduling of the IO agent relative to RT; (ii) the site and route of delivery of the immune modulator(s); (iii) the mechanism of action of the IO agent used; (iv) variation according to the TME of the specific cancer. For combination approaches, the IO agent(s) can be administered before, concurrently, or after RT.

A number of current published pre-clinical studies have attempted to address the issue of sequencing, leading to contrasting results. Young et al. showed that the administration of anti-CTLA-4 mAb prior to RT is more effective than its administration post-RT [75]. In particular, in the colorectal cancer model CT26, anti-CTLA-4 mAb, injected seven days before RT, induced better tumor responses leading to long term tumor control, compared with anti-CTLA-4 given one or five days post-irradiation. The effectiveness of the pre-treatment was partly due to T_regs_ depletion, suggesting a possible role of anti-CTLA-4 in the elimination of these cells [75]. In contrast, Twyman-Saint Victor et al., reported no difference in efficacy when anti-CTLA-4 was administered with pre- or concurrent-RT. Comparable survival and tumor growth were observed when comparing the two schedules (concurrent vs. sequential) in the B16-F10 melanoma model [82]. Thus, efficacy of scheduling may also reflect characteristics of the specific tumor type or TME.

Clinical studies have focused on the concomitant administration of RT and anti-CTLA-4. Indeed, the majority of cases reporting durable disease control or abscopal responses occurred when RT was given concurrently or immediately after therapy with anti-CTLA-4 mAb [34,36,83,84].

There is some evidence that RT leads to the induction of PD-L1 upregulation on tumor cells [31], and that combination with anti-PD-1/PD-L1 mAb improves outcome. Therefore, assessment of PD-L1 upregulation in response to RT may be informative as a predictive biomarker for the addition of anti-PD-1/PD-L1 to RT. There are some data demonstrating that the combination of anti-PD-L1/PD-1 and RT improves tumor control in breast (4T1), melanoma (4434), and colorectal (CT26) mouse models. Furthermore, concurrent administration of RT and anti-PD-L1 is more effective than anti-PD-L1 sequentially administered after the completion of RT [31,85]. Similar results have also been reported for tumor models of breast (TUBO), colon adenocarcinoma (MC38) [29], head and neck squamous cell carcinoma (B4B8, LY2) [61], and intracranial glioma (GL261) [60], where concurrent administration of RT and anti-PD-L1 demonstrated improved tumor protection (see Table 1a for the details).

In accordance with this pre-clinical observation, better outcomes were reported for NSCLC patients treated with the anti-PD-1 pembrolizumab, within 14 days of completing chemo-radiation, than those who had a delayed start to treatment with the ICI [86]. RT was also identified to increase the response rate and improve the outcome of patients treated with nivolumab in advanced NSCLC patients, when administered prior to commencing treatment with the ICI [87]. Likewise, in the TONIC trial, a subset of triple-negative breast cancer (TNBC) patients receiving a two-week pre-treatment with RT (hypofractionated regimen) before administration of nivolumab achieved clinical benefit (higher response rates and durable responses). Despite this, higher objective response rate was observed in the cohorts of patients treated with the chemotherapeutics doxorubicin and cisplatin [88]. However, the specific regimen used (RT regimen and its scheduling with nivolumab) might have hampered the full potential of RT to render the TME more responsive to the subsequent administration of nivolumab [89]. Regression was also observed for melanoma patients when anti-PD-1 was given after RT, upon failure of monotherapy approach with this ICI [90]. In a case report of melanoma, RT, followed by pembrolizumab, induced a complete response after 10 total doses of anti-PD-1 therapy [91]. A case report of metastatic Merkel cell carcinoma showed durable response under concurrent treatment of RT and the anti-PD-1 pembrolizumab [92]. Overall, the current literature suggests that concurrent or immediate sequential administration of anti-PD-1/PD-L1 after RT is most effective [85]. This scheduling approach is currently being further investigated in clinical trials in multiple different tumor types.

There is currently less data available to guide the sequencing of RT with other immunostimulatory IO agents. However, some work has been done with mAb targeting OX40, a co-stimulatory molecule belonging to the TNF receptor superfamily and expressed on CD4^+^, CD8^+^, T_regs_ and NKs [93]. Young et al. evaluated the timing of anti-OX40 mAb administration relative to RT, and demonstrated (in the colorectal cancer model CT26) that administration of this IO agent the day after radiation induced higher survival rates in mice compared to delayed (five days) or neo-adjuvant (seven days) administration, resulting in long-term tumor-specific immunity [75]. These data emphasize the importance of timing of administration of IO agents relative to RT, and the urgent requirement to study it further with preclinical models informing clinical trial design. 

### 5.2. Site and Route of Delivery

The route of delivery of IO agents is another factor that could potentially have important consequences to the generation of local and systemic anti-tumor immunity. The systemic administration of IO agents is more likely to induce systemic immune-related toxicity than intra-tumoral (I.T.) administration. Local I.T. delivery has a number of advantages: increasing tumor drug delivery, potentially generating an in-situ vaccination and decreasing the off-target irAEs associated with systemic delivery; bioavailability; and ultimately potentially increasing the therapeutic index [94].

The direct release into the TME enables high concentrations in the tumor with less systemic toxicity, which potentially facilitates the development of combination strategies involving more than one IO agent which may be better tolerated and potentially overcome resistance mechanisms associated with a single agent [95]. The release of tumor antigens via radiation, coupled with site-specific immune modulation, may lead to greater priming of T cell immunity compared to delivery via other routes, which may also improve systemic responses [95]. Furthermore, one or more sites could be injected concurrently or sequentially to boost T cell clonality, in synergy with RT, to target the possible heterogeneity of the tumor sub-clones.

A number of pre-clinical studies have directly investigated the optimal site(s) and route(s) of delivery of therapeutic co-inhibitory/co-activatory IO agents in combination with RT. Fransen et al. showed, in a colon carcinoma model (MC38), that subcutaneous and slow administration of anti-CTLA-4 mAb close to the tumor area induced tumor eradication as efficaciously as a higher dose injected systemically. The localized therapy also reduced the degree of adverse events and systemic toxicity compared to high dose systemic treatment, likely as a consequence of lower levels of antibody present in the serum. By directly comparing the two treatment routes, the authors observed that local treatment enhanced systemic response specifically mediated by CD8^+^ T cells, which were essential for the elimination of both local and distant tumors [96]. Similar results, showing comparable efficacy and reduced toxicity of peri-tumoral (p.t.) injections over i.v. (intra-venous) and s.c. (sub-cutaneous) routes, were also observed in a pre-clinical bladder carcinoma (MB49) study [97]. In particular, low-dose peri-tumoral injection of anti-CTLA-4 mAb was as effective in controlling tumor growth as the other systemic routes, and the efficacy of the local administration was enhanced by T_regs_ reduction. Furthermore, local injection reduced (ten-fold) the level of circulating antibody, interleukin-6, and overall toxicity compared to the systemic administration [97].

The safety and efficacy of I.T. delivery of ICIs and TLRs agonists in combination with RT is currently being evaluated in clinics. For example, I.T. injection of ipilimumab with local irradiation, is being investigated for the treatment of lymphoma, melanoma, colon, and rectal cancer (NCT01769222). A TLR-9 agonist administered intratumorally to B cell lymphoma [81] and follicular lymphoma patients [98] showed effective anti-tumor response. Moreover, one phase 1 trial indicated that s.c. administration of the anti-PD-1 PF-06801591, is a feasible and effective alternative to i.v. injection [99]. In addition, another study demonstrated the safety and feasibility of I.T. administration of the anti-CD40 mAb ADC-1013 in a range of metastatic solid tumors [100].

In summary, these studies confirm the feasibility of local injection of IO agents which appears to be a promising strategy for clinical application. However, further studies are required to clarify the optimal scheduling of RT and site/route of delivery of IO agents and to understand their impact on the priming, efficacy, and potency of the anti-tumor immune response, both locally and systemically.

## 6. Conclusions

The increasing awareness of the ability of RT to modulate the immune contexture of the TME, in addition to the breakthrough status of ICIs and the arrival of a new era of IO agents, have provided unprecedented opportunities to improve outcomes for cancer patients in radiation oncology. The ability of RT to potentially play a role in priming systemic anti-tumor immunity has paved the way for the optimization of strategies combining RT with IO agents to further enhance such local and systemic immunity. Although great progress and promising results have been reported with RT and IO agents in pre-clinical murine tumor models, translating these proof-of-principle efficacy studies to improving outcomes in cancer patients, is much more challenging. Clinical translation requires much greater understanding of the complex interplay between how RT interacts with immune cells and cancer cells and how this can be further modulated in combination with a particular IO agent. In order to guide the most effective way to integrate these two therapeutic modalities to improve outcomes, it will be important to investigate: (i) a range of RT dose and fractionation regimens to determine the optimal approach; (ii) the optimal RT volume and field to target; (iii) a range of different IO agent(s) to be used in combination with radiation, as we begin to think beyond ICIs to other immunomodulatory agents—such novel immunostimulatory IO agents may be aimed at “reprogramming” subpopulations of immune cells, potentially based on profiling of the individual patient TME; (iv) and finally, defining the site/route and scheduling of the IO agent(s) relative to the RT regimen, because it may have an important influence on the therapeutic outcome. In addition, it is likely that the answers to these areas of uncertainty could be very different and vary according to the tumor type and immune context, and possibly to the agent concomitantly used.

In order to make timely progress, there is an urgent requirement to investigate underlying mechanisms in pre-clinical models to deliver the “proof of principle” data required to inform clinical trial designs, and thus minimize the number of negative clinical studies. Carefully designed clinical trials “back translating” with comprehensive translational research, investigating tumor and blood immune profiling, are required. Furthermore, it will be necessary to move away from rigid well-established principles in radiation oncology based on decades of practice dictated by normal tissue repair and linear quadratic equation, to novel approaches aimed at investigating the immunomodulatory effects of RT. Such approaches may involve partial tumor irradiation or differential tumor irradiation where RT dose is varied to parts of the tumor, or using targeted approaches with stereotactic ablative radiotherapy (SABR) to multiple metastatic lesions, to investigate the optimal approach to elicit the most effective anti-tumor immune priming.

Finally, the development of predictive clinical biomarkers for RT and IO agent combinations is urgently required. Despite the enthusiasm of the radiation oncology community, with hundreds of clinical trials evaluating RT and IO agent combinations, only a small minority have associated translational research that may provide mechanistic insights in the development of RT and IO immunological biomarkers. For single agent RT, or RT in combination with IO agents, this field is still in its infancy and requires the coordinated efforts of the pioneers to incorporate biomarker-driven research into their design. Only with this focus on translational research will progress be made and provide us with the possibility that tumors may be stratified for treatment, according to immune biomarkers identified in the TME, and/or the peripheral blood, that will predict immune responses post-RT, and this potentially will inform RT and IO agent combinations. In conclusion, RT and IO agent combinations offer a unique therapeutic opportunity to improve outcomes for cancer patients receiving RT. This opportunity will only be realized if we carefully and rigorously re-evaluate the fundamental principles of radiation delivery, and consider the impact changing these parameters on radiation immune response, both locally within the TME and systemically, which may hold the key to durable tumor control.

## Figures and Tables

**Table 1:** Overview of the pre-clinical and clinical studies targeting immunomodulatory molecules in combination with RT. The tables report the main immunomodulatory molecules currently targeted in pre-clinical (**a**) and clinical (**b**) settings, their functions (co-inhibitory or co-stimulatory) and the up-to-date discoveries, listing the cancer types in which these studies were conducted in combination with RT, with particular focus on the relative immune-related responses observed. Also reported are the RT regimens and the administration scheduling/sequencing of RT–IO agent used, and that induced the immune responses indicated.

**a**							
**Targeted Molecule**	**Category**	**Cancer Type**	**Mouse Model**	**(Immune) Responses Observed**		**RT Regimen**	**RT–IO Agent Schedule**
**CTLA-4** **(Cytotoxic T Lymphocyte-** **Associated protein 4)**	Co-inhibitory	Breast cancer [25,46] Colon cancer [25]	TSA [25] 4T1 [46] MCA38 [25]	CD8^+^ T cell-mediated tumor inhibition (tumor-specific IFN-γ production); abscopal response [25]		3 × 8 Gy, 5 × 6 Gy [25]	IO agent given concurrently with RT [25]
				Survival advantage promoted by CD8^+^ T cells, with inhibition of lung metastasis development [46]		1 × 12 Gy, 2 × 12 Gy [46]	IO agent given following (1, 4, 7 days) RT [46]
**PD-1** **(Programmed Death-1)**	Co-inhibitory	Breast cancer [31,41,58,59] Colon cancer [31,41,58] Melanoma [31,41,58]	4T1 [31,58] 4T1-HA [41] CT26 [31] MC38 [58] 4434 [31] B16-OVA [41,58,59]	CD8^+^ T cells mediated immune response improving local tumor control; CD8^+^ T cells mediated long term survival and protection against tumor rechallenge; anti-tumor memory response antigen-specific; IFN-γ production by CD8^+^ T cells mediating PD-L1 upregulation on cancer cells [31]		5 × 4 Gy (4T1) [31] 5 × 2 Gy (CT26, 4434) [31]	IO agent given concurrently with RT [31]
				Antigen-specific T and B cells mediated responses; increased percentage of antigen-experienced T cells and effector memory T cells; upregulation of tumor-associated MHC; enhanced antigen-presentation in DLNs; increased tumoral T cell infiltration; improved local tumor control [41]		1 × 12 Gy [41]	IO given concurrently with RT [41]
				CD8^+^ T cells mediated effective immune response; essential role of DCs for cross-presentation/priming in tumor rejection; reduced total content of effector T cells, MDSCs and T_regs_ in both irradiated and non-irradiated sites; increased T cells intracellular expression of IFN-γ; increased expression of CD137 and PD-1 on TILs; abscopal response CD8^+^ T-cells mediated [58]		3 × 8 Gy [58]	IO given concurrently with RT [58]
		Renal cell cancer [59]	RENCA [59]	Immune response tumor-specific CD8^+^ T-cells mediated; abscopal response [59]		1 × 15Gy [59]	IO given concurrently with RT [59]
		Glioblastoma multiforme [60]	GL261-Luciferase [60]	Increased infiltration of CTLs; T_regs_ decrease; long term-survival [60]		1 × 10Gy [60]	IO given concurrently with RT [60]
**PD-L1** **(Programmed Death-Ligand 1)**	Co-inhibitory	Breast cancer [29,31] Colon cancer [29,31]	TUBO [29] 4T1 [31] CT26 [31] MC38 [29]	CD8^+^ T cell-mediated Long-term immunity; reduced local MDSCs accumulation CTL-mediated, via cytotoxic TNF-α production; PD-L1 tumoral upregulation; abscopal response [29]		1 × 12 Gy (TUBO) [29] 1 × 20 Gy (MC38) [29]	IO given concurrently with RT [29]
		Melanoma [31]	4434 [31]	CD8^+^ T cells mediated immune response improving local tumor control; CD8^+^ T cell-mediated long-term survival and protection against tumor rechallenge; anti-tumor memory response antigen-specific; IFN-γ production by CD8^+^ T cells mediating PD-L1 upregulation on cancer cells [31]		5 × 2 Gy (4434) [31]	IO agent given concurrently with RT [31]
		Head and neck squamous cell carcinoma [61]	B4B8 [61] LY2 [61]	Increased T cell infiltration; PD-L1 tumoral upregulation; enhanced tumor control; improved mice survival [61]		1 × 10 Gy [61]	IO given before (3 days), concurrently and after (2x/week until end of experiment) RT [61]
		NSCLC [62]	LLC [62]	Enhanced anti-tumor immunity mediated by infiltrated CD8^+^ T cells; reduced MDSCs accumulation and T_regs_ infiltration; increased expression of PD-L1 [62]		3 × 2 Gy [62]	IO given concurrently with RT [62]
		Pancreatic ductal adenocarcinoma [63]	KPC [63] Pan02 [63]	Enhanced infiltration of CD8^+^ T cells; increased CD8:T_regs_ ratio; reduced myeloid cells infiltration; improved tumor response [63]		1 × 12 Gy, 5 × 3 Gy, 1 × 20 Gy [63]	IO given concurrently with RT [63]
**TIGIT** **(T cell ImmunoGlobulin and ITIM domain)**	Co-inhibitory	Colon cancer [24] Melanoma [24]	CT26 [24] B16-F10 [24]	Complete anti-tumor response [24]		3 × 8 Gy [24]	IO given concurrently with RT [24]
**4-1BB** **(or CD137)**	Co-stimulatory	Breast cancer [58]	4T1 [58]	See above [58]		See above [58]	See above [58]
		Colorectal cancer [58]	MC38 [58]				
		Melanoma [58]	B16-OVA [58]				
		Glioma [64]	GL261 [64]	Anti-tumor immunity mediated by increased TILs infiltration and tumor-specific IFN-γ production; complete tumor eradication; long-term survival [64]		2 × 4 Gy [64]	IO given following (1 day) RT [64]
**TLRs** **(Toll-Like Receptors)**	Activator	Colorectal cancer [65,66]	CT26 [65,66]	CD8^+^ T cells mediated curative immune response; immune-memory tumor-specific [65]		5 × 2 Gy [65]	IO given before (1 day) or concurrently with RT [65]
		Fibrosarcoma [66]	KHT [66]	CD8^+^ T cells mediated immunity; reduction in metastatic burden; improved survival of mice; complete tumor resolution [66]		1 × 15 Gy (KHT) [66] 5 × 2 Gy (CT26) [66]	IO given before (1 h) RT [66]
		Lymphoma [67,68]	A20 [67] EG7 [67,68]	Expansion of antigen-specific CD8^+^ T cells; development of tumor-specific memory immune response; long-term tumor clearance; improved survival [67]		1 × 10 Gy (EG7) [67] 5 × 2 Gy (EL4, A20) [67]	IO given concurrently and after (1x/week for up to 5 weeks) RT [67]
			EL4 [67,68]	Long-term immune protection DCs mediated [68]		1 × 10 Gy (EL4) [68]	IO given concurrently and after (1x/week for up to 5 weeks) RT [68]
		Lung cancer [69]	3LL [69]	Specific systemic anti-tumor humoral response; augmented tumoral infiltration of NKDCs, reduced pulmonary metastasis; tumor growth inhibition; improved survival [69]		1 × 20 Gy [69]	IO given concurrently and after (2x/week for 3 weeks) RT [69]
		Melanoma [70]	B16-F1 [70] B16-F10 [70]	Enhanced systemic anti-tumor immunity via increased number of tumoral CD8^+^ T cells, reduced number of T_regs_ and MDSCs; reduced number of lung metastatic nodules; increased population of T cells expressing IFN-γ and TNF-α; decreased tumor growth [70]		1 × 2 Gy [70]	IO given before (6 h) and after (1 day) RT [70]
		Osteosarcoma [65]	LM8 [65]	See above [65]		See above [65]	See above [65]
		Renal cancer [65]	RENCA [65]				
**GITR** **(Glucocorticoid-** **Induced TNFR-Related protein)**	Co-stimulatory	Glioblastoma [71]	GL261-luciferase [71]	T cells mediated immune response; higher intra-tumoral level of CD4^+^ T cells vs. T_regs_; elevated IFN-γ and IL-2 production by CD4^+^ T cells; elevated IFN-γ and TNF-α production by CD8^+^ T cells; increased mRNA expression of M1 markers and decreased expression of M2 markers in tumor-infiltrated mononuclear cells; improved survival with a certain degree of cure rate [71]		1 × 10 Gy [71]	IO given concurrently and after (3 days) RT [71]
**CD40** **(Cluster of Differentiation 40)**	Co-stimulatory	B-cell lymphoma [72]	A31 [72] πBCL_1_ [72]	CD8^+^ T cells mediated anti-tumor immune response; long-term mice survival [72]		1 to 8 Gy (A31) [72] 1 × 5 Gy (πBCL_1_) [72]	IO given after (4 h) RT [72]
		Cervical cancer [73]	TC-1 [73]	Tumor regression; long-term survival; development of immune-memory; abscopal response [73]		1 × 6 Gy [73]	IO given after (within 3 h) RT [73]
		Pancreatic cancer [74]	KPC cells [74] Panc02 [74]	CD8^+^ T cells immune-memory; augmented T cells priming; long-term survival; abscopal effect [74]		1 × 5 Gy (Panc02) [74] 1 × 10 Gy (KPC) [74]	IO given after (within 3 h) RT [74]
**OX40** **(or CD134)**	Co-stimulatory	Colorectal cancer [75]	CT26 [75]	Long-term tumor immunity; increased survival [75]		1 × 20 Gy [75] 1 × 20 Gy [76]	IO given after (1day) RT [75]
		Lung cancer [76]	LLC-OVA [76]	CD8^+^ T cells mediated anti-tumor immunity; immune-memory; durable tumor immunity and prolonged survival [76]			IO given concurrently and after (4, 7, 10 days) RT [76]
**b**							
**Targeted Molecule**	**Category**	**Cancer Type**	**Clinical Trial (RegIstration Number and Type)**	**Trial Phase, Patients Enrolled (*n*)**	**(Immune) Responses Observed**	**RT regimen**	**RT–IO Agent Schedule**
**CTLA-4** **(Cytotoxic** **T Lymphocyte-** **Associated protein 4)**	Co-inhibitory	Melanoma [77]	NCT01557114; Interventional	Phase 1; *n*: 19	Increased CD8^+^ T cells associated with PFS; partial and complete responses observed [77]	9 Gy [77]	IO given before (week 1), concurrently (week 4) and after (weeks 7 and 10) RT [77]
		NSCLC [38]	NCT02221739; Interventional	Phase 1–2; *n*: 39	Increased level of serum IFN-β; changes in T cell clones; systemic anti-tumor response T cell-mediated [38]	5 × 6 Gy, 3 × 9.5 Gy [38]	IO given concurrently and after RT (on day 22, 43, 64 of the treatment regimen) [38]
**PD-1/PD-L1** **(Programmed Death/Ligand-1)**	Co-inhibitory	NSCLC [78]	NCT02492568; Interventional	Phase 2; *n*: 92	Increased ORR, disease control rate, median PFS and OS [78]	3 × 8 Gy [78]	IO given after (within 7 days) RT [78]
		Metastatic cancers (NSCLC, melanoma, breast cancer, pancreatic cancer, etc.) [79]	NCT02303990; Interventional	Phase 1; *n*: 24	Ki67 increase in PD-1 expressing CD8^+^ T cell; complete response and prolonged stable disease observed [79]	3 × 8 Gy [79] 1 × 17 Gy [79]	IO given before (1 week) and concurrently with RT [79]
**TLRs** **(Toll-Like Receptors)**	Activator	B cell lymphoma [80]	NCT02266147; Interventional	Phase 2; *n*: 29	Increased effector CD8^+^ and CD4^+^ T cells in the TME; decreased T-follicular helper and T_regs_ cells in the TME; tumor reduction at the treated sites; abscopal response; partial and complete responses observed [80]	2 × 2 Gy [80]	IO given concurrently and after (1×/week for 5 weeks) RT [80]
		B cell lymphoma [81]	NCT00185965; Interventional	Phase 1–2; *n:* 15	Tumor-reactive memory CD8^+^ T cells; partial and complete responses observed [81]	2 × 2 Gy [81]	IO given immediately before and after RT (and weekly for 8 additional consecutive weeks) [81]

Abbreviations. CTLs: cytotoxic T lymphocytes; Gy: gray; IL-2: interleukin-2; IO: immuno-oncology; LLC: Lewis lung carcinoma; MDSCs: myeloid derived suppressor cells; NKDCs: natural killer dendritic cells; NSCLC: non-small cell lung cancer; ORR: overall response rate; OS: overall survival; TILs: tumor infiltrating lymphocytes; PFS: progression free survival; TNF-α: tumor necrosis factor-alpha; T_regs_: regulatory T lymphocytes.

## Data Availability

No new data were created or analyzed in this study. Data sharing is not applicable to this article.

## List of Abbreviations

APCs	Antigen Presenting Cells
CAFs	Cancer Associated Fibroblasts
CTLs	Cytotoxic T Lymphocytes
CTLA-4	Cytotoxic T Lymphocyte-Associated protein-4
CTV	Clinical Target Volume
DAMPs	Damage Associated Molecular Patterns
DCs	Dendritic Cells
DLNs	Draining Lymph Nodes
ENI	Elective Nodal Irradiation
FDA	Food and Drug Administration
GITR	Glucocorticoid-Induced TNFR-Related protein
Gy	Gray
ICAMs	Intercellular Adhesion Molecules
ICAM-1	Intercellular Adhesion Molecule-1
ICD	Immunogenic Cell Death
ICI	Immune Check-point Inhibitors
IFN	Interferon
IMRT	Intensity-Modulated Radiation Therapy
IO	Immuno-Oncology
irAEs	Immune Related Adverse Events
I.T.	Intra Tumoral
i.v.	intra-venous
LLC	Lewis Lung Carcinoma
MDSCs	Myeloid Derived Suppressor Cells
MHC I	Major Histocompatibility Complex class I
MOSART	Multi-Organ Site Ablative Radiation Therapy
NKG2D	Natural Killer Group 2D
NKs	Natural Killer Cells
NSCLC	Non-Small Cell Lung Cancer
PD-1	Programmed Death-1
PD-L1	Programmed Death Ligand-1
p.t.	peri-tumoral
RT	Radiotherapy
SABR	Stereotactic ABlative Radiotherapy
s.c.	sub-cutaneous
SFRT	Spatially Fractionated RadioTherapy
TAAs	Tumor-Associated Antigens
TGF-β	Transforming Growth Factor-Beta
TIGIT	T cell ImmunoGlobulin and ITIM domain
TILs	Tumor Infiltrating Lymphocytes
TLRs	Toll-Like Receptors
TME	Tumor Microenvironment
TNF	Tumor Necrosis Factor
T_regs_	regulatory T lymphocytes
